# Genetic diversity of *Francisella tularensis* subsp. *holarctica* in Kazakhstan

**DOI:** 10.1371/journal.pntd.0009419

**Published:** 2021-05-17

**Authors:** Vladislav Shevtsov, Alma Kairzhanova, Alexandr Shevtsov, Alexandr Shustov, Ruslan Kalendar, Sarsenbay Abdrakhmanov, Larissa Lukhnova, Uinkul Izbanova, Yerlan Ramankulov, Gilles Vergnaud

**Affiliations:** 1 National Center for Biotechnology, Nur Sultan, Kazakhstan; 2 S. Seifullin Kazakh Agrotechnical University, Nur Sultan, Kazakhstan; 3 National Scientific Center for Especially Dangerous Infections named by Masgut Aykimbayev, Almaty, Kazakhstan; 4 School of Science and Technology Nazarbayev University, Nur Sultan, Kazakhstan; 5 Université Paris-Saclay, CEA, CNRS, Institute for Integrative Biology of the Cell, Gif-sur-Yvette, France; Universidad Nacional Autonoma de Mexico, MEXICO

## Abstract

Tularemia is a highly dangerous zoonotic infection due to the bacteria *Francisella tularensis*. Low genetic diversity promoted the use of polymorphic tandem repeats (MLVA) as first-line assay for genetic description. Whole genome sequencing (WGS) is becoming increasingly accessible, opening the perspective of a time when WGS might become the universal genotyping assay. The main goal of this study was to describe *F*. *tularensis* strains circulating in Kazakhstan based on WGS data and develop a MLVA assay compatible with *in vitro* and *in silico* analysis. *In vitro* MLVA genotyping and WGS were performed for the vaccine strain and for 38 strains isolated in Kazakhstan from natural water bodies, ticks, rodents, carnivores, and from one migratory bird, an Isabellina wheatear captured in a rodent burrow. The two genotyping approaches were congruent and allowed to attribute all strains to two *F*. *tularensis holarctica* lineages, B.4 and B.12. The seven tandem repeats polymorphic in the investigated strain collection could be typed in a single multiplex PCR assay. Identical MLVA genotypes were produced by *in vitro* and *in silico* analysis, demonstrating full compatibility between the two approaches. The strains from Kazakhstan were compared to all publicly available WGS data of worldwide origin by whole genome SNP (wgSNP) analysis. Genotypes differing at a single SNP position were collected within a time interval of more than fifty years, from locations separated from each other by more than one thousand kilometers, supporting a role for migratory birds in the worldwide spread of the bacteria.

## Author summary

Genotyping of *Francisella tularensis* has become a routine practice in epidemiology. Despite rapidly accumulating knowledge, the phylogeography of the pathogen is still poorly understood and discussions about geographic and temporal origins continue. One important reason is the poor characterization of the pathogen in many tularemia-endemic countries. This article describes the genetic diversity of *Francisella tularensis* subsp. *holarctica* in Kazakhstan using tandem repeat polymorphisms as well as whole genome sequencing. Thirty-nine strains were analyzed and two lineages were identified, namely B.4 and B.12. The study demonstrates a wider distribution of genotype B.4 in Asia, and identified a more basal branching point in this subclade. The obtained data support the Asian origin hypothesis for *F*. *tularensis*. The finding of identical genotypes in strains separated in time by decades and a thousand-kilometers geographic distance, confirms the ability of the bacteria for long-term preservation and fast long distances spread. The isolation of *F*. *tularensis* subsp. *holarctica* from the bird species Isabellina wheatear allows speculating about a major contribution of birds to the phylogeography of the pathogen. A genotyping protocol was developed utilizing seven polymorphic tandem repeats, two of which were identified within the framework of this work. The *in vitro* and *in silico* results are identical when using sequencing reads of 300 base-pairs or more.

## Introduction

Precise knowledge of the genetic diversity of microorganisms is useful for multiple purposes, including understanding of the emergence of pathogens, tracking and controlling their spread, as well as certifying and patenting strains of interest for biotechnologies [1–3]. DNA "fingerprinting" of bacteria began to develop with the understanding of the role of this molecule and improved throughout the development of DNA technologies. Genotyping based on DNA fragmentation and resolution by Pulsed-Field Gel Electrophoresis (PFGE), Random Amplified Polymorphic DNA (RAPD), Restriction Fragment Length Polymorphism (RFLP) have been replaced by simpler, more accurate and reproducible (numeric) methods including Multiple-Locus Variable-number of tandem-repeats (VNTR) Analysis (MLVA), MultiLocus Sequence Typing (MLST) and selected single nucleotide polymorphism (canSNP) typing [4–6]. Currently, whole-genome sequencing (WGS) of bacterial strains, the ultimate genotyping method, is becoming increasingly accessible but is still too costly especially in developing countries to allow its use as a first-line assay. In the ongoing transition period, highly discriminatory and cheap DNA based genotyping methods can be used as a first-line method for selecting strains for WGS, especially in highly endemic regions and as a low-cost assay for routine quality control of strain collections [7].

Most laboratories are using one method or more among WGS, MLST, canSNP, MLVA and/or PFGE in parallel for a number of pathogens in order to carry out epidemiological control adapted to local context, capacities, and needs [8]. In this regard, the development of *in silico* genotyping methodology based on WGS data and allowing backward compatibility is particularly useful for pathogens for which MLVA and/or MLST were considered as the “golden standard” of genotyping such as *Brucella* spp, *Bacillus anthracis*, *Yersinia pestis*, *F. tularensis* or *Neisseria meningitidis* [9–12]. Carrying out *in silico* MLST on WGS data is not difficult owing to the availability of appropriate algorithms and allows comparing the results obtained previously [13]. Obtaining a relevant *in silico* MLVA profile is a more challenging task due to the occurrence of errors in the assembly of short reads and contig breaks in VNTR locations [14]. Nevertheless, the use of appropriate sequencing reads length allows to conduct *in silico* MLVA with high reliability. It has been established for Brucella that read sizes of 200 base-pairs or more allow to correctly assemble tandem repeat arrays for most loci used in the MLVA assay and to produce a reliable MLVA profile [15]. Simple scripts have been developed to run *in silico* MLVA (https://github.com/i2bc/MLVA_finder). FASTA files of assemblies (complete genomes or contigs) are commonly used as input. However, currently available assemblers often use k-mer values shorter than read length, which can be detrimental when assembling tandem repeat arrays and does not allow to take full advantage of reads length [16].

*In silico* MLVA analysis was not previously performed for *F. tularensis*, although it is considered to be among the six most dangerous pathogens with potential for use as an agent of bioterrorism [17] and MLVA typing has been shown to be relevant [4,18]. Twenty-five VNTRs have been described so far in the *F. tularensis* genome and various schemes using a subset of these have been proposed. MLVA correctly classifies *F. tularensis* into the three subspecies, of which the most pathogenic for humans is *F. tularensis* subsp. *tularensis* common in North America with a single strain of unclear origin described in Europe [18,19]. *F. tularensis* subsp. *holarctica* distributed in most of the northern hemisphere is less pathogenic for humans [20,21]. *F. tularensis* subsp. *mediasiatica* present in Central Asian region and southern Siberia has not been associated with human infections [22]. *F. novicida* was once proposed as subspecies based on its high genetic similarity (97%) with *F. tularensis* but this assignment is controversial and confusing in terms of ecology, evolution, and metabolism [23,24]. *F. novicida* detected in North America and Australia is weakly virulent and can cause disease in people with immunosuppression [23,25]. The evolution of *F. tularensis* is clonal [26] and the species constitute a monophyletic lineage in contrast to *F. novicida* which is a clear outgroup with a very different behavior [24]. The geographic origin and the date of emergence of *F. tularensis* are unknown, and the population structure is reflecting a capacity to travel long distances, as well as a very low mutation rate [26]. The clonal evolution of *F. tularensis* allows to use selected single nucleotide polymorphisms commonly called canSNPs [27] to robustly designate sublineages. Subspecies holarctica is currently subdivided in four clades identified by canSNPs, B.4, B.6, B.12 and B.16 (alias japonica). B.4 is the dominant holarctica lineage in North America, B.6 is dominant in Western Europe, whereas B.12 is dominant across Asia and Eastern Europe [5]. Currently available data on the B.6 holarctica sublineage in Western Europe is suggesting that Western Europe was contaminated from Eastern Europe [26,28,29]. The most basal B.16 lineage has been reported in Japan, China [30,31] and Turkey [32]. This feature combined with the geographic distribution of mediasiatica which was found only in Central Asia [22] is supporting an Asian or Central Asian origin for *F. tularensis* [30].

Due to the endemicity of tularemia in Kazakhstan, extensive field work has been done during the past seventy years. This work has allowed to map a number of natural foci accross the country [33]. Natural reservoirs of tularemia are registered in twelve among the 14 administrative regions of Kazakhstan and the total area of the reservoirs is about a fifth of the country’s territory (552 thousand square kilometers). During the past 90 years (1928-2018) about 10.000 human cases of tularemia were registered in Kazakhstan. The majority of cases occured in the 1950s and were caused by labor migration of unvaccinated populations to endemic areas [34]. In the 1928-1970s period, the majority of cases occured as outbreaks involving from 105 to 1791 cases. Improving sanitary conditions and routine vaccination reduced the tularemia incidence to sporadic cases [35,36]. Nevertheless, *F. tularensis* is present in East Kazakhstan, Akmola, West Kazakhstan, Aktobe, North Kazakhstan and Pavlodar regions, as shown by periodic epizootics in rodents [37]. Strains of *F. tularensis* subsp. *holarctica* are routinely isolated from at least 26 vertebrate species including humans, rodents, hares and birds as well as invertebrate vectors (blood-sucking insects and arthropods) from natural habitats across Kazakhstan. Also, strains of *F. tularensis* subsp. *mediasiatica* are isolated in the Almaty and Zhambyl regions from rodents, hares and invertebrate vectors.

There is however currently little information about the genetic diversity of circulating strains. The present report is a contribution in order to fill this gap. We applied whole genome sequencing as well as *in vitro* MLVA genotyping on a relevant subset of 39 strains isolated in Kazakhstan. We compared the *in vitro* and *in silico* MLVA data deduced from WGS data, as well as the clustering achieved by MLVA and whole genome SNP (wgSNP) analysis. A panel of seven VNTR loci which can be amplified in a single PCR reaction and is well adapted to the genetic diversity observed in Kazakhstan was defined in order to provide a first-line genotyping assay usable on a large scale. In conclusion, we describe the genetic diversity of *F. tularensis* subsp. *holarctica* determined by MLVA, canSNP and wgSNP methods and compare with currently available WGS data of worldwide origins. This is the first report of WGS data for holarctica strains from Kazakhstan.

## Materials and methods

### Strain and DNA extraction

A total of 39 strains of *F. tularensis* subsp. *holarctica* were obtained from the Kazakhstan “Republican collection of microorganisms and the depository of pathogens of especially dangerous infections” of the Republican State Enterprise on the Right of Economic Management “National Scientific Center for Especially Dangerous Infections named by Masgut Aykimbayev” of the Ministry of Healthcare of the Republic of Kazakhstan (NSCEDI). Thirty-eight strains were isolated from rodents (14 strains), ticks (19 strains), carnivores (two strains), one migratory bird and two water samples from eight regions of Kazakhstan in the period from 1951 to 2018. The vaccine strain *F. tularensis* 15 NIIEG (alias LVS) originating from Kazakhstan was included as a reference strain (Table 1). The strains were isolated during routine monitoring or during periods of epizootological outbreaks, in accordance with applicable laws. All vertebrate hosts but one were trapped, and tick were host-seeking, pasture ticks collected from the vegetation. Work with cultures of tularemia pathogens was carried out in accordance with current regulatory and methodological documents on the organization and conduct of laboratory diagnosis of tularemia in Kazakhstan.

**Table 1:** Characterization of *F. tularensis* subsp. *holarctica* strains from Kazakhstan.

Key	Region within Kazakhstan	Landscape*	Coordinates	Date of isolation	Host**
Tul-106_KZ	West-Kazakhstan	S	49°34’ 49°26’	12.12.2014	*Microtus arvalis* (corpse found)
Tul-11_KZ	East Kazakhstan	B	47°51’ 84°28’	01.06.2001	Tick
Tul-112_KZ	Pavlodar	S	52°22’ 78°58’	15.06.2016	Tick - *Haemaphysalis concinna*
Tul-128_KZ	West-Kazakhstan	S	48°50’ 49°52’	18.02.2015	*Meriones tamariscinus*
Tul-13_KZ	West-Kazakhstan	S	49°26’ 49°48’	30.01.2012	*Mus musculus*
Tul-132_KZ	Aktobe	S	50°50’ 54°53’	29.05.2017	*Mustela nivalis*
Tul-133_KZ	West-Kazakhstan	S	49°31’ 50°34’	21.12.2012	*Mus musculus*
Tul-135_KZ	Almaty	B	45°36’ 80°28’	15.07.1953	(water)
Tul-139_KZ	Akmola	S	51°47’ 68°19’	1955	Tick - *Dermacentor*
Tul-149_KZ	West-Kazakhstan	S	51°1’ 49°55’	23.05.2013	Tick - *D*. *marginatus*
Tul-15_KZ	Aktobe	S	50°50’ 54°53’	15.05.2017	*Mustela nivalis*
Tul-151_KZ	Aktobe	S	49°42’ 55°3’	10.05.2011	*Spermophilus pygmaeus*
Tul-153_KZ	West-Kazakhstan	S	49°35’ 53°6’	24.05.2011	*Oenanthe isabellina*
Tul-154_KZ	Almaty	S	46°13’ 78°54’	27.08.1975	Tick - *Dermacentor*
Tul-155_KZ	East Kazakhstan	S	49°54’ 82°45’	1951	*Arvicola amphibius*
Tul-161_KZ					(vaccine strain 15 NIIEG)
Tul-17_KZ	East Kazakhstan	S	49°54’ 82°45’	1951	*Arvicola amphibius*
Tul-18_KZ	Almaty	B	45°37’ 80°26’	1953	(water)
Tul-19_KZ	West-Kazakhstan	S	49°23’ 51°24’	25.01.2017	*Apodemus uralensis*
Tul-2_KZ	Aktobe	S	49°27’ 54°51’	22.05.2016	Tick
Tul-20_KZ	Pavlodar	S	51°8’ 50°18’	07.09.2016	Tick
Tul-30_KZ	West-Kazakhstan	S	49°15’ 49°28’	03.12.2015	*Mus musculus*
Tul-52_KZ	Aktobe	S	48°27’ 61°23’	22.04.2013	*Allactaga major*
Tul-6_KZ	West-Kazakhstan	S	50°25’ 52°33’	18.10.2012	*Mus musculus*
Tul-61_KZ	Aktobe	S	49°13’ 54°33’	27.03.2010	Tick – *D*. *pictus*
Tul-66_KZ	Almaty	S	45°47’ 81°20’	29.05.2004	Tick - *Dermacentor*
Tul-67_KZ	Karaganda	S	48°40’ 71°39’	1989	Tick
Tul-68_KZ	Almaty	S	45°19’ 82°18’	28.04.2002	Tick - *Dermacentor*
Tul-7_KZ	West-Kazakhstan	S	49°27’ 49°48’	29.09.2004	Tick
Tul-71_KZ	Almaty	T	45°12’ 74°44’	17.05.1963	Tick - *D*. *daghestanicus*
Tul-76_KZ	Almaty	B	45°23’ 80°3’	10.05.2018	Tick - *D*. *marginatus*
Tul-78_KZ	West-Kazakhstan	S	49°49’ 53°46’	19.04.2007	Tick
Tul-86_KZ	Karaganda	S	49°12’ 73°3’	1986	Tick - *D*. *marginatus*
Tul-87_KZ	Zhambyl	T	44°49’ 71°9’	2004	Tick - *D*. *daghestanicus*
Tul-92_KZ	West-Kazakhstan	S	51°1’ 54°1’	09.07.1988	Tick - *D*. *marginatus*
Tul-93_KZ	West-Kazakhstan	S	51°37’ 52°18’	23.01.2004	*Mus musculus*
Tul-97_KZ	Pavlodar	S	52°28’ 78°9’	30.05.2013	Tick - *Haemaphysalis conсinna*
Tul-98_KZ	West-Kazakhstan	S	49°29’ 50°30’	19.12.2011	*Cricetulus migratorius*
Tul-99_KZ	West-Kazakhstan	S	49°5’ 50°10’	24.05.2011	*Spermophilus major*

* S, Floodplain swamp; B, Foothill brook; T, Turgay.

** All ticks were pasture ticks, collection from vegetation. Vertebrate hosts were trapped except when indicated otherwise (one instance).

The lyophilized strains were plated on transparent agar nutrient medium for cultivation and isolation of tularemia microbe with vitamins and mineral additives (“FT-agar", FBIS SRCAMB, Obolensk, Russia) and cultured for 48 hours at a temperature of 37 ± 1°C. A bacterial suspension was prepared and inactivated by the addition of merthiolate sodium to a final concentration of 0.01%, heated for 30 min at a temperature of 56°C. DNA was isolated from inactivated suspension with QIAamp DNA Mini Kit (Qiagen, Germany).

### Genome sequencing, single nucleotide polymorphism (SNP) calling and canSNP assignment

Sequencing libraries were prepared using Nextera XT DNA Library Preparation Kit (Illumina, USA). Sequencing was performed using MiSeq Reagent Kit v3 (600 cycles) on the Illumina MiSeq platform. Sequencing quality was checked using FastQC v0.11.15 [38]. High-quality sequence selection was performed using MultiQC v3 [39]. Raw reads can be accessed from the European Nucleotide Archive [7] under study accession PRJNA639508 (https://www.ebi.ac.uk/ena/browser/view/PRJNA639508; run accessions SRR13617503-SRR13617541).

SNPs were called by mapping raw sequencing reads on a reference genome using BioNumerics version 7.6.3 (Applied-Maths, Laethem-Saint-Martin, Belgium) with default parameters as previously described except when mapping Ion Torrent PGM sequence data for which the “allow gapped alignment” option was activated [22]. Publicly available assemblies and raw reads were downloaded via EBI-ENA (last download on August 25^th^ 2020). The list of public assemblies and sequence reads archives evaluated for this report is provided in S1 Table, as well as the list of accessions used for each figure. Assemblies were split into 50 bp long artificial reads with a 10X cover and no error model. These artificial reads were then mapped on the reference genome for SNP calling as done with raw sequencing reads. BioNumerics was used with default parameters (no resampling) for Maximum Parsimony analysis [40] and dendrogram drawing.

Raw reads datasets were assembled using SPAdes [41] version 3.13.1 and SKESA [42] version 2.3.0. The canSNP assignment of each strain was deduced from genome assemblies using CanSNPer2 (https://github.com/FOI-Bioinformatics/CanSNPer2) [43] and the available *F. tularensis* canSNPer2 scheme [18,28,43,44].

### *In vitro* MLVA typing

*In vitro* MLVA genotyping was performed with the previously described 25 VNTR loci [4]. The ten most variable loci (Ft-M02, Ft-M03, Ft-M04, Ft-M05, Ft-M06, Ft-M10, Ft-M20, Ft-M22, Ft-M23 and Ft-M24) were analysed using capillary electrophoresis with fluorescent primers (Applied Biosystems genetic analyzers) [18]. The remaining 15 loci (Ft-M01, Ft-M07, Ft-M08, Ft-M09, Ft-M11, Ft-M12, Ft-M13, Ft-M14, Ft-M15, Ft-M16, Ft-M17, Ft-M18, Ft-M19, Ft-M21 and Ft-M25) were typed using monoplex PCR and agarose gel electrophoresis [22]. The resulting MLVA data were deposited in the public *Francisella tularensis* MLVA database accessible at https://microbesgenotyping.i2bc.paris-saclay.fr/.

### *In silico* MLVA on genome assemblies and on raw sequence reads

*In silico* MLVA was run on genome assemblies by applying the MLVA_finder.py script with default settings (https://github.com/i2bc/MLVA_finder) and primers listed in S2 Table.

The size of the polymorphic VNTR arrays being smaller than the 300 bp reads produced here, we also typed raw reads data. A simple script based on bbduk (BBTools, https://sourceforge.net/projects/bbmap/) was developed to identify reads containing VNTRs and both flanking sequences (https://github.com/Vladislav-Shevtsov/search-primers-in-reads/). Primers were selected that were located as close as possible to the VNTR loci (S2 Table). When selecting primers for *in silico* instead of *in vitro* detection, attention was paid only to uniqueness and to the formation of only the target fragment. The script combines all reads containing a VNTR locus of interest in a single multifasta file on which the MLVA_finder.py script can subsequently be applied.

### Identification of additional VNTR loci

The search for additional VNTR markers was performed using Tandem Repeats Finder, version 4.07b [45] and microsatellite repeats finder (http://insilico.ehu.es/mini_tools/microsatellites/). Tandem repeats were searched in *F. tularensis* subsp. *tularensis* SCHU S4 substr. NR-28534 (assembly accession GCA_000628925.1) and *F*. *tularensis* subsp. *holarctica* FTNF002-00 (assembly accession GCF_000017785.1). New VNTR loci showing two alleles or more among the twelve genomes *F. tularensis* subsp. *holarctica* (NZ_CP010289, NC_019551, NC_017463, NZ_CP010288 and NC_009749), *F. tularensis* subsp. *mediasiatica* (NC_010677), *F. novicida* (NC_008601), *F. tularensis* subsp. *tularensis* (NC_006570, NC_016933, NZ_CP009753, NZ_CP012037 and NC_009257) were further investigated *in silico* on 276 assemblies of *F. tularensis* deposited in EBI-ENA. The discriminatory power of each locus was evaluated using Hunter-Gaston Discrimination Index (HGDI) [46].

## Results

### Whole genome SNP analysis

**Fig 1:**
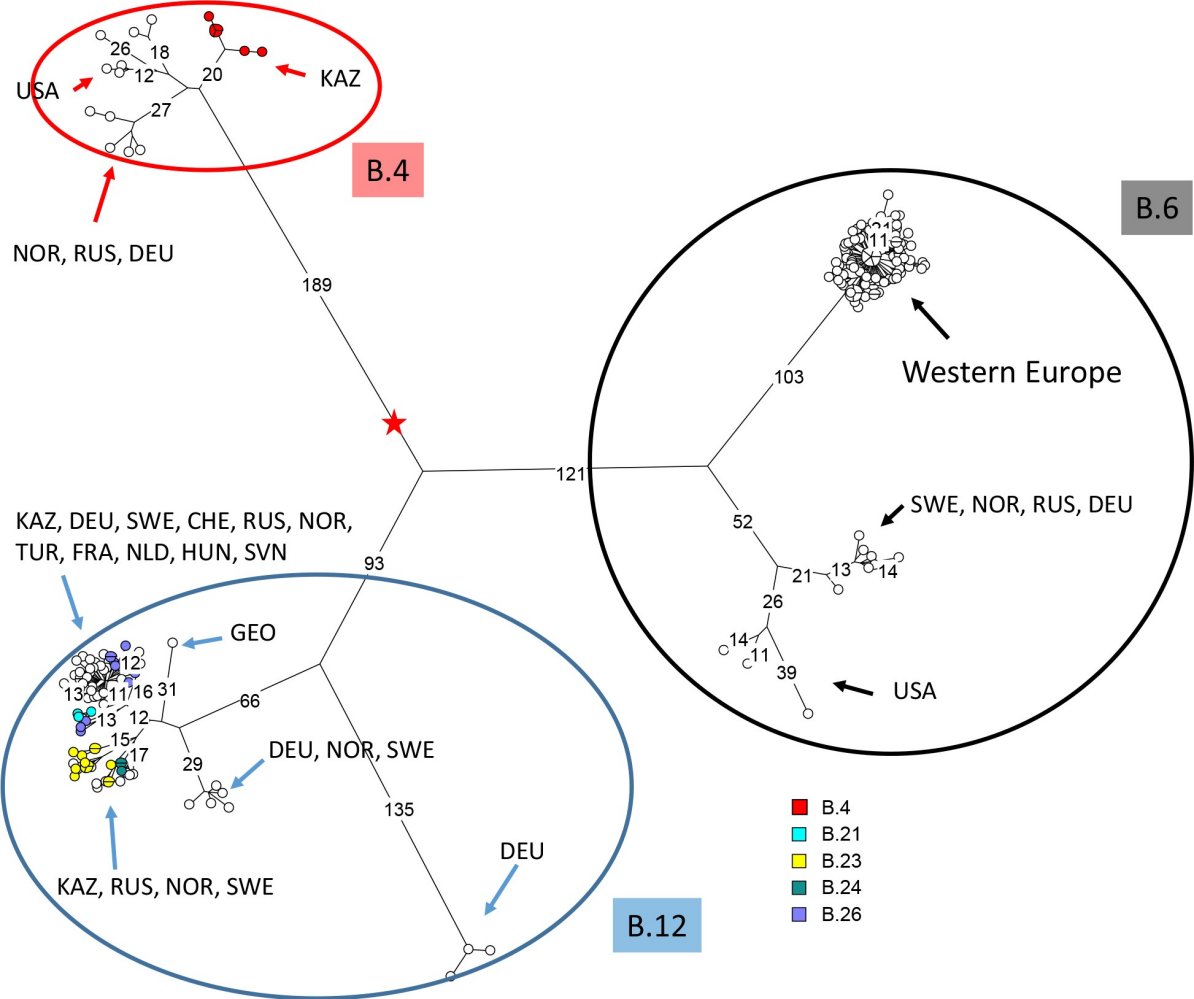
Positioning the Kazakhstan strains within the *holarctica* subspecies by wgSNP and Maximum Parsimony analysis. Two thousand and twenty-two SNPs were called among 219 strains comprising 39 strains from Kazakhstan and 180 public data sets belonging to *holarctica* B.4, B.6 or B.12 (B.16 alias japonica strains were used to root the tree and are not shown). The red star shows the position of the MRCA. Branch lengths of more than ten SNPs are indicated. The branch scale is linear. The total tree size is 2034 (homoplasia 0.6%). The circles representing strains from Kazakhstan are colored according to canSNP assignment as indicated. The geographic origin of strains constituting secondary sublineages is indicated using three-letters country codes.

The WGS data obtained from the 39 strains was used for wgSNP sequence phylogenetic analysis together with representative publicly available WGS data (S1 Table). Fig 1 shows the population structure of the *holarctica* subspecies excluding the most basal *holarctica* bv. japonica lineage (currently designated B.16). The three sublineages, B.4, B.6 and B.12 are clearly separated. The distances from the root indicated by the red star to the tips are very similar. Each of the three sublineages is itself divided in a limited number of distinct sublineages. These sublineages show a strong geographic association with the exceptions of B.12 sublineages B.23 and particularly B.42. In agreement with current knowledge regarding its geographic distribution, B.6 is not detected in Kazakhstan in the present collection [28].

Six strains isolated in 1951-1989 in East Kazakhstan, Akmola, Almaty and Karaganda regions define a new basal lineage within *holarctica* clade B.4 (Figs 1 and S1). The rest of the B.4 group is split in two subgroups, one showing a strong geographic association with North-America, and the other with Russia and Western Europe (Norway and Germany). Branch expansions from the B.4 most recent common ancestor (MRCA) to the tips are similar and vary from a minimum 46 SNPs up to 56 SNPs (S1 Fig).

**Fig 2:**
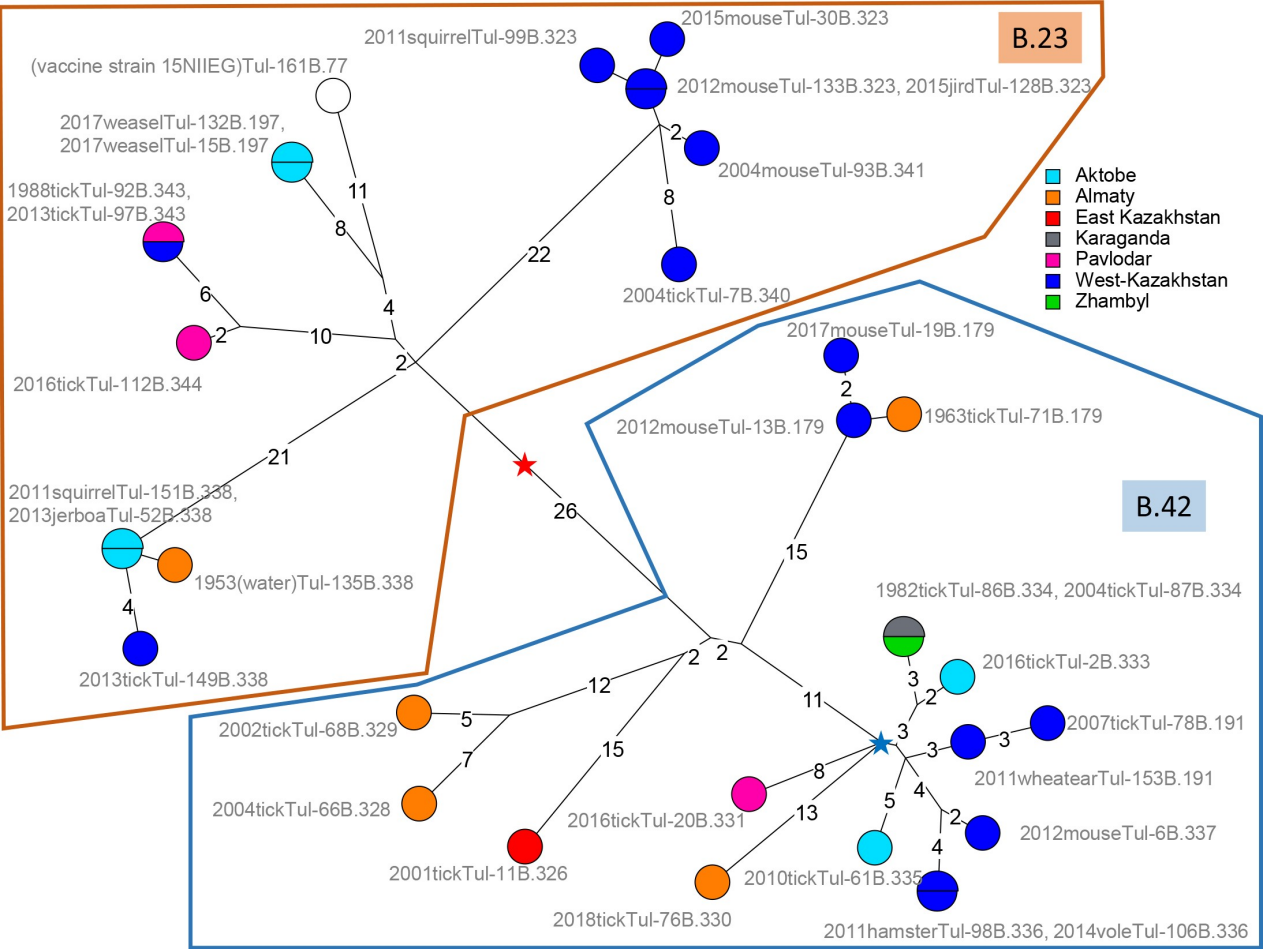
Focus on the 33 Kazakhstan strains belonging to sublineages B.23 and B.42. Two hundred and fifty-four SNPs were called by mapping on reference SCHU_S4 (assembly accession GCA_000008985). The Maximum Parsimony tree size is 254 (no homoplasy). The red star indicates the position of the MRCA. The blue star indicates the root of the B.66 polytomy shown in a larger context in S2 Fig. Strains are labelled with year of isolation, host, strain Id and CanSNP assignment. The color code reflect the geographic origin within Kazakhstan (region level). Branch lengths longer than one are indicated. The branch scale is linear.

All the other strains belong to *holarctica* clade B.12. Sixteen belong to subclade B.12_B.23 and seventeen to subclade B.12_B.42 [47]. Fig 2 illustrates that identical whole genome SNP genotypes are observed in strains collected in distant locations at different times. Strain Tul-92_KZ collected in West Kazakhstan in 1988 is identical to Tul-97_KZ collected in Pavlodar in 2013, i.e. twenty-five years later more than one thousand kilometers away. A single SNP separates Tul-13_KZ collected in 2012 in West-Kazakhstan from Tul-71_KZ collected 49 years earlier in the Almaty region, or Tul-52_KZ isolated in 2013 in Aktobe from Tul-135_KZ isolated sixty years earlier in the Almaty region. In both instances, the most recent strain showed the ancestral genotype, and the single SNP in the derived genotype is a G to A transition, in agreement with the previously observed AT mutation bias [26]. This indicates that identical wgSNP profile can be perpetuated for twenty-five years and strengthens previous observation of identical wgSNP genotypes in strains collected in Sweden 15 years apart [48] and in France, Switzerland and Germany up to 31 years apart [28]. Some sub-branches show a strong geographic homogeneity, in particular a group of six B.23 strains isolated in West Kazakhstan in years 2004-2015. They constitute a three-branches polytomy up to eight SNPs in length. The closest strain (seven up to 14 SNPs away) among public WGS data is strain NO-18/2011 isolated in Norway in 2011 (S1 Table).

In contrast, eleven strains coming from six regions within Kazakhstan constitute a polytomy with three branches radiating from the blue star within the B.42 sublineage (Fig 2). S2 Fig includes all publicly available WGS data assigned to this polytomy. In terms of canSNP nomenclature, the polytomy belongs to B.12 B.42 and includes all branches subordinate to B.66. The 64 strains assigned to the B.66 polytomy have been collected between 1928 and 2018 (S2 Fig and S1 Table) and define four branches arbitrarily labelled here L1 to L4. From the MRCA of the polytomy indicated by the red star in S2 Fig to the tips, distances are in the range 5-19 SNPs. Branch L1 in the B.66 polytomy is represented by a single strain, B-8364 isolated in the Far East of Siberia in 1966. Similarly branch L3 is represented by strain Tul-76 from Kazakhstan. L4 with 48 strains is by far the most represented in available WGS data. In addition to strain Tul-20 from Kazakhstan and strain B-7558 presumably from Russia, it comprises strains from Western Europe, particularly Germany (29 strains) and including Scandinavia. Within Europe, Sweden appears to have a special status, as strains from Sweden are present also in L2, and contribute one basal branch within L4. This observation is in agreement with previous reports of a relatively high genetic diversity in Scandinavia, which might suggest that Scandinavia is the primary source for tularemia in Western Europe [47] or that Scandinavia provides more ecological opportunities for long-term maintenance of *F*. *tularensis* lineages. Regarding polytomy B.66, Central Asia appears to be a better candidate for being the source since all four branches are represented.

Both lineages L2 and L4 contain secondary polytomies (S2 Fig). The secondary polytomy in L2 contains six branches, with length from root (blue star) to tips varying from five to 14 SNPs. This polytomy is remarquable by its geographic diversity. Two branches are represented by strains isolated in Sweden, and two branches are constituted by strains from West Kazakstan. One of them was isolated from a migratory bird (wheatear *Oenanthe isabellina*) [49]. The main polytomy in L4 (purple star) contains eleven radiating branches and is shown with a more specific country color code in S3 Fig. Eight branches are represented by strains from Germany and two by strains from Switzerland. The last branch is the most diverse, and was observed in Scandinavia, France, Hungaria and Germany. The most parcimonious interpretation of available data is that Scandinavia (possibly Sweden) was contaminated once by each of the two lineages, L2 and L4, possibly via migratory birds and that secondary contaminations occurred from Scandinavia towards Western Europe, mainly Germany. Much more will need to be known regarding the implication of migratory birds [50] in order to evaluate this preliminary hypothesis.

### *In vitro* genotyping by MLVA

In order to develop a first line genotyping assay, we evaluated the discriminatory power of MLVA in the present collection. Five among the 25 VNTR loci constituting the full MLVA typing scheme are polymorphic, Ft-M3, Ft-M4, Ft-M6, Ft-M20 and Ft-M22 (Tables 2 and S3). The highest discrimination was observed at the FT-M3 locus in agreement with previous reports. Twelve alleles and an HGDI of 0.90 were observed among the 39 strains. Ft-M6 locus was the second most variable, with four alleles. At the Ft-M4, Ft-M20 and Ft-M22 loci, three, two and two alleles were observed respectively.

**Table 2:** Discrimination of VNTR loci for strains circulating in Kazakhstan.

Locus name	Number of alleles/genotypes	HGDI (95% confidence interval)
Ft-M3	12	0.9042 (0.8730,0.9354)
Ft-M4	3	0.3104 (0.1416,0.4792)
Ft-M6	4	0.6073 (0.4904,0.7242)
Ft-M20A	2	0.1457 (0.0013,0.2902)
Ft-M22	2	0.2672 (0.1072,0.4272)
**MLVA5 Nur-Sultan**	**18**	**0.9325 (0.8950,0.9700)**
insilico-FT-4	2	0.0513 (0.0000,0.1473)
insilico-FT-8	2	0.2672 (0.1072,0.4272)
**MLVA7 Nur-Sultan**	**19**	**0.9406 (0.9074,0.9738)**

The five polymorphic loci among the classic 25 VNTR resolve 18 genotypes in the present collection (MLVA5 Nur-Sultan, Table 2). Two additional VNTR loci polymorphic in the present collection were identified by *in silico* analysis, insilico-FT-4_6bp_97bp_2u and insilico-FT-8_4bp_152bp_3u (S2 Table). According to *in silico* reads analysis, they are polymorphic in the present collection and allow to resolve one additional genotype (Tables 2 and S3). All seven loci can be amplified in a single PCR reaction with different fluorescent dyes and analysed on a genetic analyzer in one run.

### *In silico* MLVA for *F. tularensis* subsp. *holarctica* isolates from Kazakhstan

To satisfy future needs in terms of compatibility between *in silico* and *in vitro* MLVA genotyping we evaluated two different approaches to deduce the MLVA genotype from sequence data. In the first and more traditional approach, sequencing reads were assembled before analysis. Two popular assemblers were compared, SPAdes and SKESA. In the second approach, raw sequencing reads corresponding to the VNTR loci were extracted and directly analysed to produce the *in silico* MLVA genotype. The approach takes advantage of the 300 base-pairs reads produced which should be long enough to enclose in a subset of reads the whole tandem repeat and a few flanking nucleotides from both sides, even for the largest Ft-M3 alleles.

The results obtained by direct reads analysis were fully identical to *in vitro* data (S3 Table). In contrast, discrepancies were observed in the assemblies. SKESA failed to assemble the Ft-M6 allele in two strains. Ft-M3 was not assembled in eleven and five strains with SPAdes and SKESA respectively (S3 Table). Furthermore Ft-M3 SPAdes assemblies showed an incorrect size in five additional strains. SKESA assemblies predicted an incorrect size for insilico-FT-4 in all strains but one.

In summary, direct analysis of 300 bp reads performed significantly better than assemblies analysis. As expected, the number of recovered reads is proportionally dependent on the size of the VNTR allele as illustrated for the Ft-M3 VNTR in S4 Table.

### Clustering of *F. tularensis* subsp. *holarctica* strains using MLVA and congruence with canSNP

**Fig 3:**
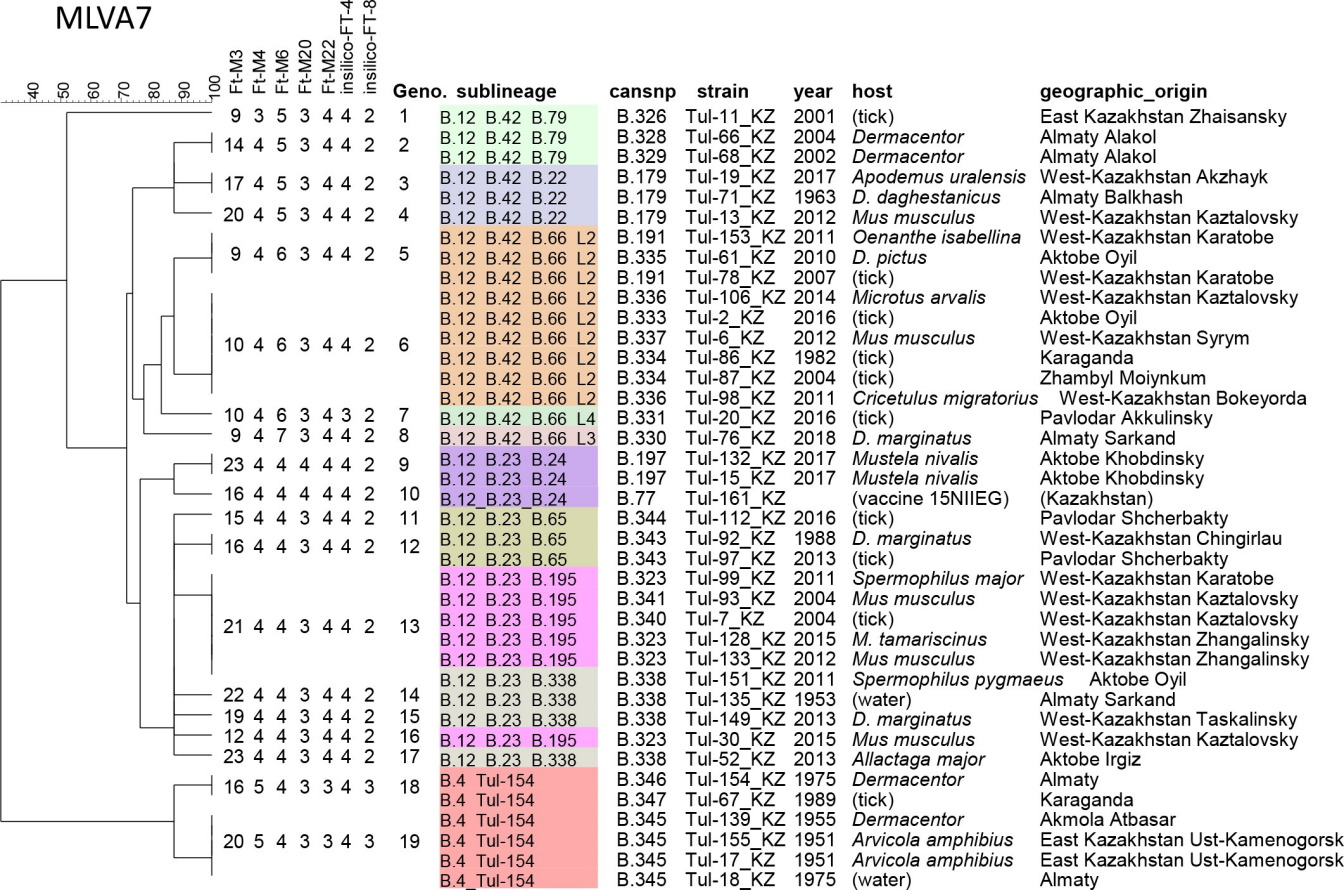
MLVA clustering of the 39 Kazakhstan strains. The MLVA data for the seven polymorphic loci was clustered using the UPGMA method. The main canSNP sublineages are colored.

Based on seven variable loci, the 39 strains were clustered into 19 genotypes (Fig 3). Ten genotypes are unique, five genotypes are shared by two strains. The four most frequent genotypes are shared by 3, 4, 6 and 6 strains respectively. The eleven strains belonging to the B.66 polytomy are correctly clustered. More generally, the B.4, B.12_B.23 and B.12_B.42 groups are globally clustered as expected from the wgSNP analysis. Some phylogenetic inconsistencies of terminal branches where MLVA is not congruent with wgSNP phylogeny are visible, due to the homoplasia inherent to tandem repeat polymorphism. Most of these MLVA terminal branches are defined only by the highly variable Ft-M3 locus.

## Discussion

We present the results of genotyping of thirty-nine *F. tularensis* subsp. *holarctica* strains isolated in Kazakhstan including the vaccine strain 15 NIIEG, using MLVA, canSNP and wgSNP. MLVA genotyping was performed by the classical genotyping scheme including 25 loci [4]. In agreement with previous reports five among the subset of ten loci selected by Vogler et al. were variable among the analyzed strains [4,18,51]. For genotyping of isolates circulating in the Central Asian region, we propose a MLVA7 panel combining these five classical VNTR loci and two additional VNTRs identified by *in silico* analysis of public WGS data. The proposed *in silico* MLVA genotyping script based on the direct analysis of 300 bp long raw reads made it possible to correctly identify all alleles in the 39 strains. Consequently, MLVA7 constitutes a one PCR assay compatible with *in silico* WGS analysis which should facilitate the selection of strains for sequencing and the quality control of strain identity.

Based on wgSNP analysis, four genetic groups are distinguished in *F. tularensis* subsp. *holarctica*: B.4, B.6, B.12 and B.16 [20,44,52]. In our study, six strains isolated during 1951-89 in Eastern and Central Kazakhstan were assigned to the B.4 genetic group. Strains belonging to the B.4 genetic group were previously found in North America, China, and Northern-Eastern Europe [18,20,31,53,54]. The B.4 genotype might be relatively prevalent in Asia and the sequenced isolates from Kazakhstan allow to define a more basal branching point. This and the presence of B.16 subclade in Japan, Turkey, Tibet and Australia increase the likelihood that *F. tularensis* subsp. *holarctica* has an Asian origin [30]. The remaining 33 strains from Kazakhstan were assigned to the B.12 genetic group, which is widely distributed in Central to Eastern Europe [29,52].

Tularemia has been and still is the subject of important monitoring in Kazakhstan because of its zoonotic potential. A number of natural foci have been characterised in Kazakhstan and elsewhere, however the global ecology and phylogeography of this important pathogen remains poorly understood [55]. For instance, although the strict clonality of the evolution of the causal agent predicts that it emerged in one location in space and time, the geographic origin of *F*. *tularensis* is unknown. One reason for this situation is that in spite of major progress achieved in the past fifteen years owing to the emergence of powerful genotyping tools, and important efforts in terms of WGS production, data is missing for many countries. Another reason is that the phylogeography of *F*. *tularensis* is characterised by the finding of almost identical strains in very distant places and/or at very different dates [26,28,54]. A third reason is the poor knowledge of *F*. *tularensis* closest neighbors including *F*. *novicida*. It is the knowledge of closest neighbors which provided the strongest argument for an African origin of both *Mycobacterium tuberculosis* and *B*. *anthracis* [56,57].

The work presented here is confirming the remarkable features of the phylogeography of *F*. *tularensis*, in particular the growing evidence in favor of the capacity of the bacteria for long-term maintenance combined with a capacity for long-distance spread. This would suggest that *F*. *tularensis* has a capacity of «perpetuation», i.e. long-term maintenance without genetic changes, in a state implying a low level of replication [58]. A water-borne viable but non-culturable state has been proposed to explain long-term maintenance [59,60]. However, the available evidence is still fragile and limited. Also this behavior does not seem to fit with the relatively homogeneous branch length from root to tips observed in *F*. *tularensis* [22]. Data from many geographic areas is lacking and in addition, most investigations so far were restrospective analyses of historical collection. The analyses required the reculturing of the strains to prepare new DNA extractions. One cannot formally exclude cross-contaminations and additional studies will be required to confirm the present observations. In particular, it would be highly recommended to prepare a DNA batch for later sequencing purposes at the time of the very first isolation and growth of the pathogen. This DNA batch could be validated by MLVA7 *in vitro* genotyping and the resulting data could be archived in an online *F*. *tularensis* MLVA database. This initial genotype could serve as a DNA identification control of the eventual WGS data which could be *in silico* genotyped at least if sequencing reads are longer than 300 bp.

Interestingly, owing to the extensive field sampling in natural foci regularly done in Kazakhstan, a strain was recovered from an Isabellina wheatear incidentally trapped out of a rodent burrow in West Kazakhstan [49]. Although birds have been suspected as potential spreaders of *F*. *tularensis* [61], we found very little direct evidence in the litterature involving birds, either migratory [49,50] or scavenger [62]. The Isabellina wheatear is a migratory insectivorous bird. In spring, it lay eggs in burrows of rodents. Between March and August, birds will visit a number of burrows. After that, winter migrations will start, and rodents will come back to burrows to hibernate. Some fleas are shared between wheatear and rodents [63,64]. The Isabellina wheatear breeding geographic distribution ranges from North-East Greece to North East China. It winters in Africa, the Arabian Peninsula and India where it shares wintering areas with the Northern wheatear (*Oenanthe oenanthe)*, which has an even more widespread geographic range, including Scandinavia, Alaska, North-East Canada. A potential role of the Isabellina wheatear in the propagation of plague has previously been proposed [64,65].

Such an implication would help explain some aspects of the phylogeography of the bacteria, including long-distance transportation and long term preservation (within the soil ecosystem of burrows). This would imply that the birds are healthy carriers. Birds would be infected in their breeding area, and would contaminate other migratory birds, such as the Northern wheatear, in their feeding grounds. The implication of migratory birds might also provide a working hypothesis regarding the dating of the emergence of the clonal *F*. *tularensis*. It would have followed the end of the last glaciation peak about 20000 years ago. The Isabelline wheatear is considered to have arrived in the lower Volga region approximately 10000 years ago (reviewed in [64]).

## Supporting information

S1 Fig Focus on the B.4 lineage. The WGS data from the 17 strains belonging to B.4 were mapped on reference genome OSU18 (assembly accession GCA_000014605) for SNP identification. Two hundred and sixty-six SNPs were called, the Maximum Parsimony tree has a size of 269 (homoplasia 1.12%). For each strain, the country of origin (three letters code), year of isolation, host, strain Id and canSNP assignment are indicated when known. Coloring reflects sublineage assignment. Branches longer than one are labelled with size. The blue star indicates the position of the MRCA.
(TIF)

S2 Fig Phylogenetic analysis of wgSNP data of 64 strains belonging to the four branches B.12/B.42/B.66 polytomy. Three hundred and twenty-five SNPs were identified by mapping on the FSC200 genome sequence (assembly accession GCA_000168775). The Maximum Parsimony tree has a size of 325 (no homoplasy). Branch length is proportional to number of SNPs and ranges from one SNP up to 14. The red star indicates the position of the MRCA of the polytomy. Four branches arbitrarily labelled L1 to L4 radiate from the red star. Distances from the red star to the tips vary from five (L4 strain 4534-12-01 isolated in 2012 in Germany) up to 19 (L4 strain 14T0052 isolated in 2014 in Germany) SNPs. Circles are labelled with year of isolation and strain Id, and colored according to geographic origin as indicated.
(TIF)

S3 Fig Focus on the eleven branches polytomy within B.12/B.42/B.66/L4. The polytomy comprises 20 strains isolated in Europe, predominantly Germany, in the years 2000-2018. Ninety-six SNPs were identified by mapping on the FSC200 genome sequence (assembly accession GCA_000168775). The Maximum Parsimony tree has a size of 96 (no homoplasy). Branch length from the MRCA indicated by the purple star to the tips varies from two up to eleven SNPs. Circles are labelled with the three-letters country code, year of isolation, strain Id and host. They are colored according to geographic origin.
(TIF)

S1 Table List of assemblies and sequence reads evaluated. The “map_SCHU_S4_percent” column indicates for each dataset the percentage of the SCHU_S4 genome (assembly accession GCA_000008985) which does not contribute to the SNP search. This percentage corresponds to regions deleted in the strain being mapped, and also to repeated sequences. The “remarks” column provides a list of duplicate entries (usually strains for which both assemblies and raw data have been deposited, and also reference strains which have been independently sequenced). Entries labelled as “redundant” are entries showing an identical wgSNP profile with an other entry. The last four columns indicate which public datasets were used for the making of the indicated figures.
(XLSX)

S2 Table Primers for *in silico* MLVA7. (XLSX)

S3 Table MLVA results obtained by *in vitro* and different *in silico* methods. (XLSX)

S4 Table Number of raw reads containing the Ft-M3 locus and flanking primers (as indicated in S2 Table) detected in the 39 sequence reads datasets in view of the repeat copy number and dataset size. (XLSX)

## References

[1] Savin C, Criscuolo A, Guglielmini J, Le Guern A-S, Carniel E, Pizarro-Cerdá J, et al. Genus-wide *Yersinia* core-genome multilocus sequence typing for species identification and strain characterization. Microbial genomics. 2019;5(10). 10.1099/mgen.0.000301 31580794 PMC6861861

[2] Dunne WM Jr, Pouseele H, Monecke S, Ehricht R, van Belkum A. Epidemiology of transmissible diseases: array hybridization and next generation sequencing as universal nucleic acid-mediated typing tools. Infection, Genetics and Evolution. 2018;63:332–45. 10.1016/j.meegid.2017.09.019 28943408

[3] Terletsky VP, Tyshchenko VI, Novikova II, Boikova IV, Tyulebaev SD, Shakhtamirov IY. An efficient method for genetic certification of *Bacillus subtilis* strains, prospective producers of biopreparations. Microbiology+. 2016;85(1):71–6. 10.1134/S0026261716010136 WOS:000370791300008. 27301128

[4] Johansson A, Farlow J, Larsson P, Dukerich M, Chambers E, Byström M, et al. Worldwide genetic relationships among *Francisella tularensis* isolates determined by multiple-locus variable-number tandem repeat analysis. Journal of bacteriology. 2004;186(17):5808–18. 10.1128/JB.186.17.5808-5818.2004 15317786 PMC516809

[5] Vogler AJ, Birdsell D, Price LB, Bowers JR, Beckstrom-Sternberg SM, Auerbach RK, et al. Phylogeography of *Francisella tularensis*: global expansion of a highly fit clone. J Bacteriol. 2009;191(8):2474–84. Epub 2009/03/03. 10.1128/JB.01786-08 19251856 PMC2668398

[6] Mitchell CL, Andrianaivoarimanana V, Colman RE, Busch J, Hornstra-O’Neill H, Keim PS, et al. Low cost, low tech SNP genotyping tools for resource-limited areas: Plague in Madagascar as a model. PLoS Negl Trop Dis. 2017;11(12):e0006077. Epub 2017/12/12. 10.1371/journal.pntd.0006077 29227994 PMC5739503

[7] Shevtsova E, Vergnaud G, Shevtsov A, Shustov A, Berdimuratova K, Mukanov K, et al. Genetic diversity of *Brucella melitensis* in Kazakhstan in relation to World-wide diversity. Front Microbiol. 2019;10:1897. 10.3389/fmicb.2019.01897 31456793 PMC6700508

[8] Jensen M, Schjørring S, Björkman J, Torpdahl M, Litrup E, Nielsen E, et al. External quality assessment for molecular typing of *Salmonella* 2013–2015: performance of the European national public health reference laboratories. European Journal of Clinical Microbiology & Infectious Diseases. 2017;36(10):1923–32. 10.1007/s10096-017-3015-7 28573470 PMC5602099

[9] Scholz H, Vergnaud G. Molecular characterisation of *Brucella* species. Rev Sci Tech. 2013;32(1):149–62. 10.20506/rst.32.1.2189 23837373

[10] Levy H, Fisher M, Ariel N, Altboum Z, Kobiler D. Identification of strain specific markers in *Bacillus anthracis* by random amplification of polymorphic DNA. FEMS microbiology letters. 2005;244(1):199–205. 10.1016/j.femsle.2005.01.039 15727841

[11] Antwerpen MH, Prior K, Mellmann A, Höppner S, Splettstoesser WD, Harmsen D. Rapid high resolution genotyping of *Francisella tularensis* by whole genome sequence comparison of annotated genes (“MLST+”). PLoS One. 2015;10(4):e0123298. 10.1371/journal.pone.0123298 25856198 PMC4391923

[12] Rouphael NG, Stephens DS. *Neisseria meningitidis*: biology, microbiology, and epidemiology. Neisseria meningitidis: Springer; 2012. p. 1–20. 10.1007/978-1-61779-346-2_1 PMC4349422 21993636

[13] Kimura B. Will the emergence of core genome MLST end the role of *in silico* MLST? Food microbiology. 2018;75:28–36. 10.1016/j.fm.2017.09.003 30056960

[14] Nadon C, Van Walle I, Gerner-Smidt P, Campos J, Chinen I, Concepcion-Acevedo J, et al. PulseNet International: Vision for the implementation of whole genome sequencing (WGS) for global food-borne disease surveillance. Euro surveill. 2017;22(23):30544. 10.2807/1560-7917.ES.2017.22.23.30544 28662764 PMC5479977

[15] Vergnaud G, Hauck Y, Christiany D, Daoud B, Pourcel C, Jacques I, et al. Genotypic expansion within the population structure of classical *Brucella* species revealed by MLVA16 typing of 1404 *Brucella* isolates from different animal and geographic origins, 1974–2006. Frontiers in microbiology. 2018;9:1545. 10.3389/fmicb.2018.01545 30050522 PMC6052141

[16] Ambroise J, Irenge LM, Durant J-F, Bearzatto B, Bwire G, Stine OC, et al. Backward compatibility of whole genome sequencing data with MLVA typing using a new MLVAtype shiny application for *Vibrio cholerae*. PloS one. 2019;14(12):e0225848. 10.1371/journal.pone.0225848 31825986 PMC6905556

[17] Rotz LD, Khan AS, Lillibridge SR, Ostroff SM, Hughes JM. Public health assessment of potential biological terrorism agents. Emerging infectious diseases. 2002;8(2):225. 10.3201/eid0802.010164 11897082 PMC2732458

[18] Vogler AJ, Birdsell D, Wagner DM, Keim P. An optimized, multiplexed multi-locus variable-number tandem repeat analysis system for genotyping *Francisella tularensis*. Lett Appl Microbiol. 2009;48(1):140–4. Epub 2008/11/21. 10.1111/j.1472-765X.2008.02484.x . 19018964

[19] Guryčová D. First isolation of *Francisella tularensis* subsp. *tularensis* in Europe. European journal of epidemiology. 1998;14(8):797–802. 10.1023/a:1007537405242 9928875

[20] Appelt S, Köppen K, Radonić A, Drechsel O, Jacob D, Grunow R, et al. Genetic diversity and spatial segregation of *Francisella tularensis* subspecies *holarctica* in Germany. Front Cell Infect Microbiol. 2019;9:376. Epub 2019/11/30. 10.3389/fcimb.2019.00376 31781515 PMC6851236

[21] Samrakandi M, Zhang C, Zhang M, Nietfeldt J, Kim J, Iwen PC, et al. Genome diversity among regional populations of *Francisella tularensis* subspecies *tularensis* and *Francisella tularensis* subspecies *holarctica* isolated from the US. FEMS Microbiol Lett. 2004;237(1):9–17. 10.1016/j.femsle.2004.06.008 15268932

[22] Timofeev V, Bakhteeva I, Titareva G, Kopylov P, Christiany D, Mokrievich A, et al. Russian isolates enlarge the known geographic diversity of *Francisella tularensis* subsp. *mediasiatica*. PLoS One. 2017;12(9):e0183714. 10.1371/journal.pone.0183714 28873421 PMC5584958

[23] Kingry LC, Petersen JM. Comparative review of *Francisella tularensis* and *Francisella novicida*. Front Cell Infect Microbiol. 2014;4:35. Epub 2014/03/25. 10.3389/fcimb.2014.00035 24660164 PMC3952080

[24] Johansson A, Celli J, Conlan W, Elkins KL, Forsman M, Keim PS, et al. Objections to the transfer of *Francisella novicida* to the subspecies rank of *Francisella tularensis*. Int J Syst Evol Microbiol. 2010;60(Pt 8):1717–8. Epub 2010/08/07. 10.1099/ijs.0.022830-0 20688748 PMC7442299

[25] Whipp MJ, Davis JM, Lum G, de Boer J, Zhou Y, Bearden SW, et al. Characterization of a novicida-like subspecies of *Francisella tularensis* isolated in Australia. Journal of medical microbiology. 2003;52(9):839–42. 10.1099/jmm.0.05245-0 12909664

[26] Dwibedi C, Birdsell D, Larkeryd A, Myrtennas K, Ohrman C, Nilsson E, et al. Long-range dispersal moved *Francisella tularensis* into Western Europe from the East. Microb Genom. 2016;2(12):e000100. Epub 2017/03/30. 10.1099/mgen.0.000100 28348839 PMC5359409

[27] Van Ert MN, Easterday WR, Huynh LY, Okinaka RT, Hugh-Jones ME, Ravel J, et al. Global genetic population structure of *Bacillus anthracis*. PLoS One. 2007;2(5):e461. Epub 2007/05/24. 10.1371/journal.pone.0000461 17520020 PMC1866244

[28] Kevin M, Girault G, Caspar Y, Cherfa MA, Mendy C, Tomaso H, et al. Phylogeography and Genetic Diversity of *Francisella tularensis* subsp. *holarctica* in France (1947-2018). Front Microbiol. 2020;11:287. Epub 2020/03/21. 10.3389/fmicb.2020.00287 32194525 PMC7064806

[29] Koene M, Rijks J, Maas M, Ruuls R, Engelsma M, van Tulden P, et al. Phylogeographic Distribution of Human and Hare *Francisella Tularensis* Subsp. *Holarctica* Strains in the Netherlands and Its Pathology in European Brown Hares (*Lepus Europaeus*). Front Cell Infect Microbiol. 2019;9:11. Epub 2019/02/26. 10.3389/fcimb.2019.00011 30805312 PMC6378916

[30] Lu Y, Yu Y, Feng L, Li Y, He J, Zhu H, et al. Phylogeography of *Francisella tularensis* from Tibet, China: Evidence for an asian origin and radiation of holarctica-type Tularemia. Ticks Tick Borne Dis. 2016;7(5):865–8. Epub 2016/05/07. 10.1016/j.ttbdis.2016.04.001 . 27150591

[31] Wang Y, Peng Y, Hai R, Xia L, Li H, Zhang Z, et al. Diversity of *Francisella tularensis* subsp. *holarctica* lineages, China. Emerg Infect Dis. 2014;20(7):1191–4. Epub 2014/06/26. 10.3201/eid2007.130931 24963721 PMC4073844

[32] Kilic S, Birdsell DN, Karagoz A, Celebi B, Bakkaloglu Z, Arikan M, et al. Water as Source of *Francisella tularensis* Infection in Humans, Turkey. Emerg Infect Dis. 2015;21(12):2213–6. Epub 2015/11/20. 10.3201/eid2112.150634 26583383 PMC4672436

[33] Kunitsa T, Sadovskaya V, Izbanova U. [Current state of epidemiological monitoring of tularemia in natural foci of Kazakhstan]. In: Aikimbayev A, editor. Collection of works on tularemia dedicated to the 100th anniversary of doctor of medical Sciences, Professor Masgut Aikimbayev. Almaty: Kazakh scientific center for quarantine of zoonotic infections named after M. Aikimbayev; 2016. p. 190–211. Russian. 10.1039/c5cp06476g

[34] Meka-Mechenko T, Aikimbayev A, Kunitza T, Ospanov K, Temiralieva G, Dernovaya V, et al. Clinical and epidemiological characteristic of tularemia in Kazakhstan. Przeglad epidemiologiczny. 2003;57(4):587–91. 15029832

[35] Kunitsa T, Izbanova U, Meka-Mechenko T, Yakupov V. [The modern clinical and epidemical peculiarities of manifestation of tularemia in Kazakhstan urbanized territories]. Life without danger Health Prevention Longevity. 2013;8(2):41–6. Russian.

[36] Kunitsa T, Meka-Mechenko T, Lukhnova L. [Incidence of tularemia in Kazakhstan]. Probl especially dangerous infections 2001;1:52. Russian.

[37] Izbanova U, Kunitsa T, Meka-Mechenko T, Kazakov S, Kyraubayev K, Ayazbayev T. [Epizootic and epidemic situation on tularemia in Kazakhstan in 2011–2012]. Disinfection Antiseptic 2013;4(2):28–31. Russian.

[38] Andrews S. FastQC: a quality control tool for high throughput sequence data. Babraham Bioinformatics, Babraham Institute, Cambridge, United Kingdom; 2010.

[39] Ewels P, Magnusson M, Lundin S, Käller M. MultiQC: summarize analysis results for multiple tools and samples in a single report. Bioinformatics. 2016;32(19):3047–8. 10.1093/bioinformatics/btw354 27312411 PMC5039924

[40] Fitch WM. Toward defining the course of evolution: minimum change for a specific tree topology. Systematic Zoology. 1971;20(4):406–16.

[41] Bankevich A, Nurk S, Antipov D, Gurevich AA, Dvorkin M, Kulikov AS, et al. SPAdes: a new genome assembly algorithm and its applications to single-cell sequencing. J Comput Biol. 2012;19(5):455–77. 10.1089/cmb.2012.0021 22506599 PMC3342519

[42] Souvorov A, Agarwala R, Lipman DJ. SKESA: strategic k-mer extension for scrupulous assemblies. Genome biology. 2018;19(1):153. 10.1186/s13059-018-1540-z 30286803 PMC6172800

[43] Lärkeryd A, Myrtennäs K, Karlsson E, Dwibedi CK, Forsman M, Larsson P, et al. CanSNPer: a hierarchical genotype classifier of clonal pathogens. Bioinformatics. 2014;30(12):1762–4. 10.1093/bioinformatics/btu113 24574113

[44] Wittwer M, Altpeter E, Pilo P, Gygli SM, Beuret C, Foucault F, et al. Population Genomics of *Francisella tularensis* subsp. *holarctica* and its Implication on the Eco-Epidemiology of Tularemia in Switzerland. Front Cell Infect Microbiol. 2018;8:89. Epub 2018/04/07. 10.3389/fcimb.2018.00089 29623260 PMC5875085

[45] Benson G. Tandem repeats finder: a program to analyze DNA sequences. Nucleic acids research. 1999;27(2):573–80. 10.1093/nar/27.2.573 9862982 PMC148217

[46] Hunter PR, Gaston MA. Numerical index of the discriminatory ability of typing systems: an application of Simpson’s index of diversity. Journal of clinical microbiology. 1988;26(11):2465–6. 10.1128/JCM.26.11.2465-2466.1988 3069867 PMC266921

[47] Karlsson E, Svensson K, Lindgren P, Bystrom M, Sjodin A, Forsman M, et al. The phylogeographic pattern of *Francisella tularensis* in Sweden indicates a Scandinavian origin of Eurosiberian tularaemia. Environ Microbiol. 2013;15(2):634–45. Epub 2012/12/21. 10.1111/1462-2920.12052 . 23253075

[48] Johansson A, Larkeryd A, Widerstrom M, Mortberg S, Myrtannas K, Ohrman C, et al. An outbreak of respiratory tularemia caused by diverse clones of *Francisella tularensis*. Clin Infect Dis. 2014;59(11):1546–53. Epub 2014/08/07. 10.1093/cid/ciu621 25097081 PMC4650766

[49] Kdyrsikh BG, Sundukov RI, Kuspanov AK, Kdyrsikhova GG, Ayazbaev TZ, Mikanov NS. About the isolation of the tularemia agent from the Isabelline wheatear (*Oenanthe isabellina*) in West Kazakhstan region. Quarantinable and zoonotic infections in Kazakhstan. 2013;28(2):71–2.

[50] de Carvalho IL, Zé-Zé L, Alves AS, Pardal S, Lopes RJ, Mendes L, et al. *Borrelia garinii* and *Francisella tularensis* subsp. holarctica detected in migratory shorebirds in Portugal. Eur J Wildl Res. 2012;58:857–61 10.1007/s10344-012-0617-3

[51] Ariza-Miguel J, Johansson A, Fernández-Natal MI, Martínez-Nistal C, Orduña A, Rodríguez-Ferri EF, et al. Molecular investigation of tularemia outbreaks, Spain, 1997-2008. Emerg Infect Dis. 2014;20(5):754–61. Epub 2014/04/23. 10.3201/eid2005.130654 24750848 PMC4012790

[52] Gyuranecz M, Birdsell DN, Splettstoesser W, Seibold E, Beckstrom-Sternberg SM, Makrai L, et al. Phylogeography of *Francisella tularensis* subsp. *holarctica*, Europe. Emerg Infect Dis. 2012;18(2):290–3. Epub 2012/02/07. 10.3201/eid1802.111305 22305204 PMC3310461

[53] Sissonen S, Rossow H, Karlsson E, Hemmilä H, Henttonen H, Isomursu M, et al. Phylogeography of *Francisella tularensis* subspecies *holarctica* in Finland, 1993-2011. Infect Dis (Lond). 2015;47(10):701–6. Epub 2015/05/26. 10.3109/23744235.2015.1049657 . 26004621

[54] Myrtennäs K, Marinov K, Johansson A, Niemcewicz M, Karlsson E, Byström M, et al. Introduction and persistence of tularemia in Bulgaria. Infect Ecol Epidemiol. 2016;6:32838. Epub 2016/10/30. 10.3402/iee.v6.32838 27790972 PMC5084392

[55] Telford SR 3rd, Goethert HK. Ecology of *Francisella tularensis*. Annu Rev Entomol. 2020;65:351–72. Epub 2019/10/11. 10.1146/annurev-ento-011019-025134 . 31600457 PMC8300880

[56] Vergnaud G. *Bacillus anthracis* evolutionary history: taking advantage of the topology of the phylogenetic tree and of human history to propose dating points. Erciyes Med J. 2020;42(4):362–9. Epub 21/04/2020. 10.14744/etd.2020.64920

[57] Blouin Y, Hauck Y, Soler C, Fabre M, Vong R, Dehan C, et al. Significance of the identification in the Horn of Africa of an exceptionally deep branching *Mycobacterium tuberculosis* clade. PLoS One. 2012;7(12):e52841. Epub 2013/01/10. 10.1371/journal.pone.0052841 23300794 PMC3531362

[58] Telford SR 3rd, Goethert HK. Toward an understanding of the perpetuation of the agent of tularemia. Front Microbiol. 2010;1:150. Epub 2010/01/01. 10.3389/fmicb.2010.00150 21687803 PMC3109306

[59] Hennebique A, Boisset S, Maurin M. Tularemia as a waterborne disease: a review. Emerg Microbes Infect. 2019;8(1):1027–42. Epub 2019/07/10. 10.1080/22221751.2019.1638734 31287787 PMC6691783

[60] Forsman M, Henningson EW, Larsson E, Johansson T, Sandstrom G. *Francisella tularensis* does not manifest virulence in viable but non-culturable state. FEMS Microbiol Ecol. 2000;31(3):217–24. Epub 2000/03/17. 10.1111/j.1574-6941.2000.tb00686.x . 10719202

[61] Splettstoesser WD, Mätz-Rensing K, Seibold E, Tomaso H, Al Dahouk S, Grunow R, et al. Re-emergence of *Francisella tularensis* in Germany: fatal tularaemia in a colony of semi-free-living marmosets (*Callithrix jacchus*). Epidemiol Infect. 2007;135(8):1256–65. Epub 2007/02/20. 10.1017/S0950268807008035 17306050 PMC2870702

[62] Padeshki PI, Ivanov IN, Popov B, Kantardjiev TV. The role of birds in dissemination of *Francisella tularensis*: first direct molecular evidence for bird-to-human transmission. Epidemiol Infect. 2010;138(3):376–9. Epub 2009/08/12. 10.1017/S0950268809990513 . 19664305

[63] Burdelov AS, Kassenova AK. [Contacts of isabelline wheatear and its specific fleas with the plague pathogen]. Quarantinable and zoonotic infections in Kazakhstan. 2001;(4):17–9. Russian.

[64] Popov NV, Sludsky AA, Zavialov EV, Udovikov AI, Tabachishin VG, Anikin VV, et al. [A probable role of *Oenanthe isabellina* and other birds in the plague enzootic mechanism]. Povolzhsky journal of ecology. 2007;(3):215–26. Russian.

[65] Balakhonov SV, Korzun VM, Verzhutsky DB, Mikhaylov EP, Rozhdestvensky EN, Denisov AV. The First Case of *Yersinia pestis* subsp. *pestis* Isolation in the Territory of the Altai Mountain Natural Plague Focus. Communication 2. Probable Ways and Mechanisms of Plague Agent Main Subspecies Importation into the Territory of the Focus. Problems of Particularly Dangerous Infections. 2013;(2):5–10. 10.21055/0370-1069-2013-2-5-10

