# Supplementary figures and images for: Genetic diversity of Francisella tularensis subsp. holarctica in Kazakhstan
Source: PLoS Negl Trop Dis. 2021 May 17;15(5):e0009419. doi: 10.1371/journal.pntd.0009419 (PMC8158875; doi:10.1371/journal.pntd.0009419)

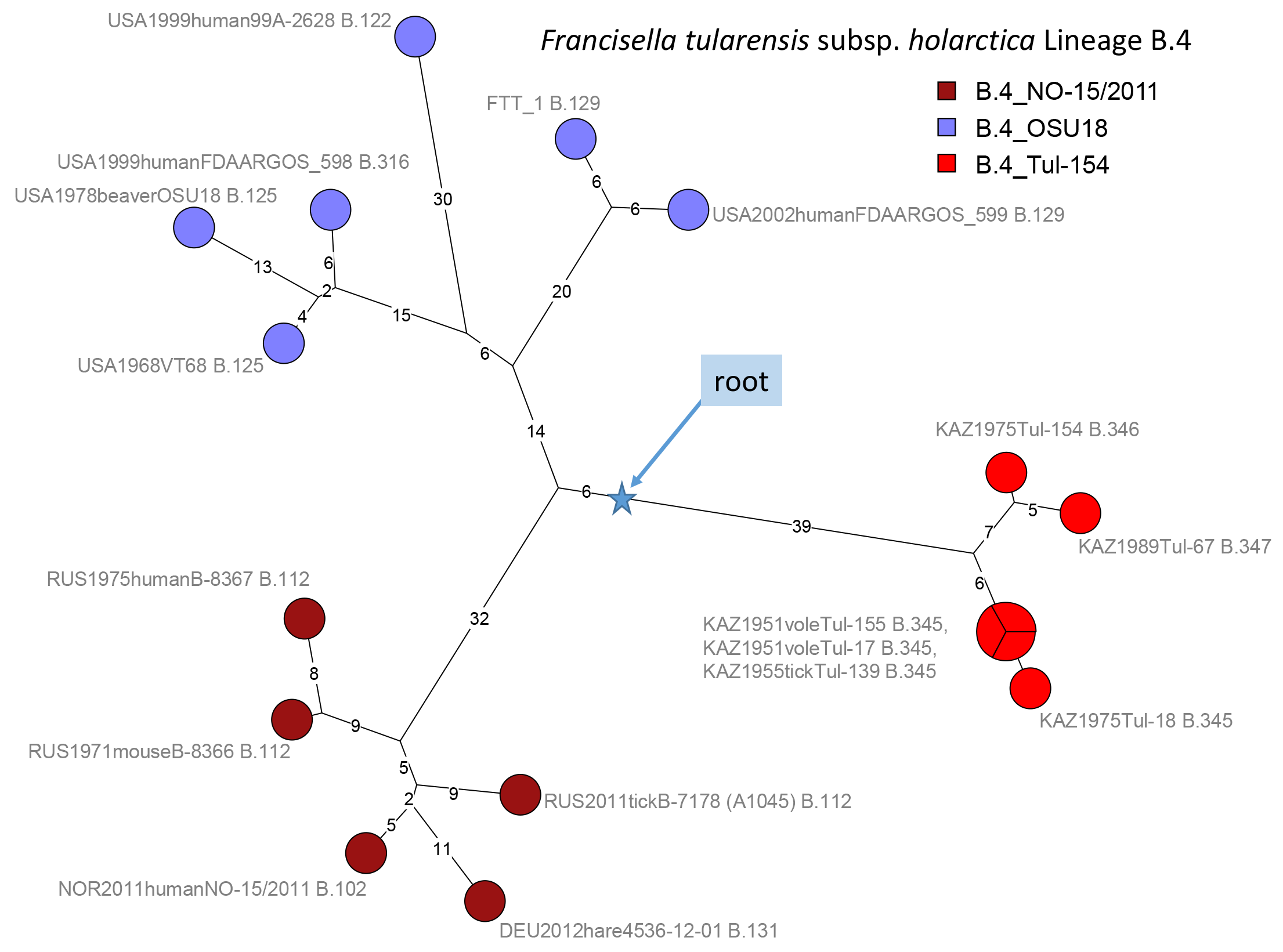

Supplement: S1 Fig — The WGS data from the 17 strains belonging to B.4 were mapped on reference genome OSU18 (assembly accession GCA_000014605) for SNP identification. Two hundred and sixty-six SNPs were called, the Maximum Parsimony tree has a size of 269 (homoplasia 1.12%). For each strain, the country of origin (three letters code), year of isolation, host, strain Id and canSNP assignment are indicated when known. Coloring reflects sublineage assignment. Branches longer than one are labelled with size. The blue star indicates the position of the MRCA. (TIF) [file pntd.0009419.s001.tif]

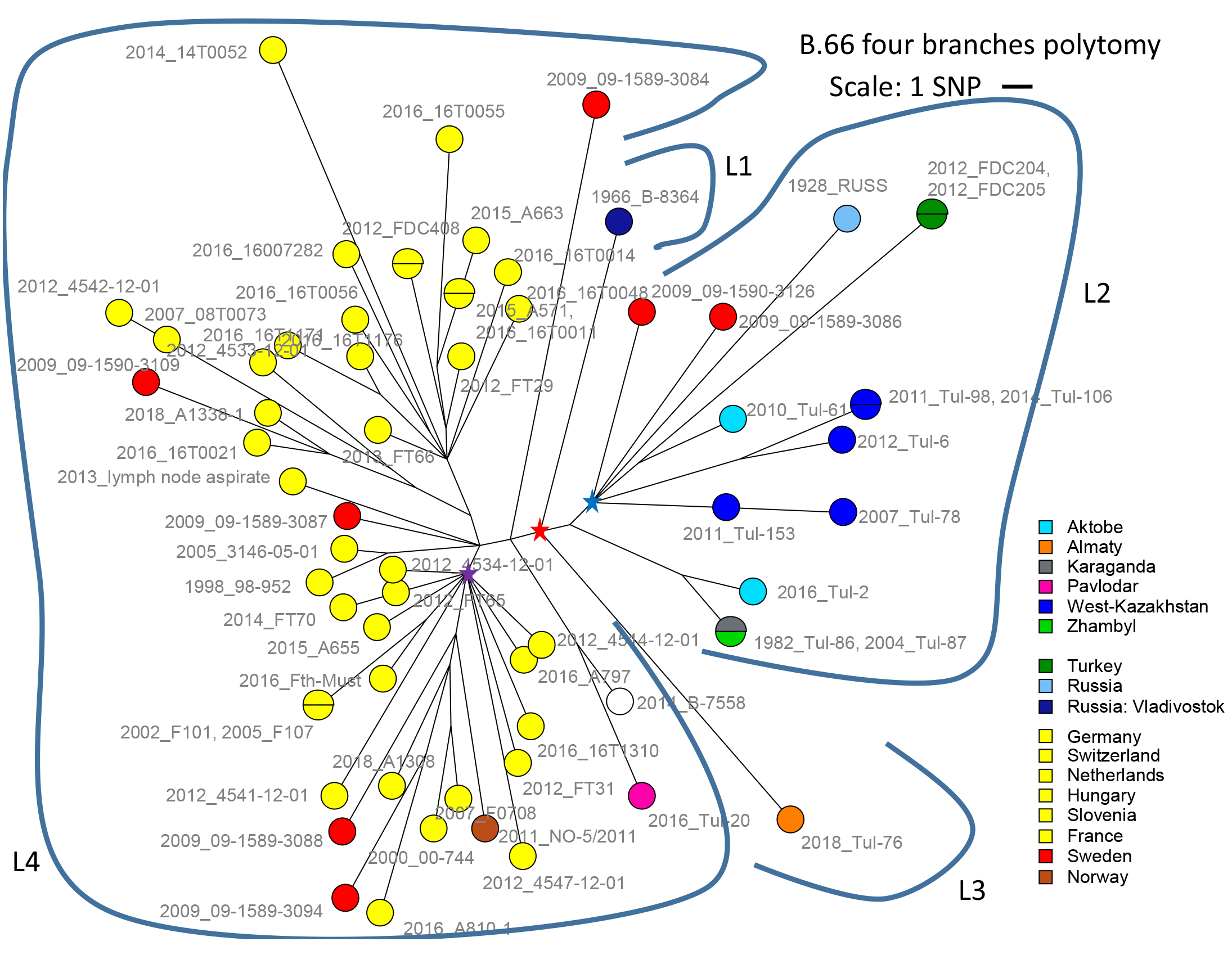

Supplement: S2 Fig — Three hundred and twenty-five SNPs were identified by mapping on the FSC200 genome sequence (assembly accession GCA_000168775). The Maximum Parsimony tree has a size of 325 (no homoplasy). Branch length is proportional to number of SNPs and ranges from one SNP up to 14. The red star indicates the position of the MRCA of the polytomy. Four branches arbitrarily labelled L1 to L4 radiate from the red star. Distances from the red star to the tips vary from five (L4 strain 4534-12-01 isolated in 2012 in Germany) up to 19 (L4 strain 14T0052 isolated in 2014 in Germany) SNPs. Circles are labelled with year of isolation and strain Id, and colored according to geographic origin as indicated. (TIF) [file pntd.0009419.s002.tif]

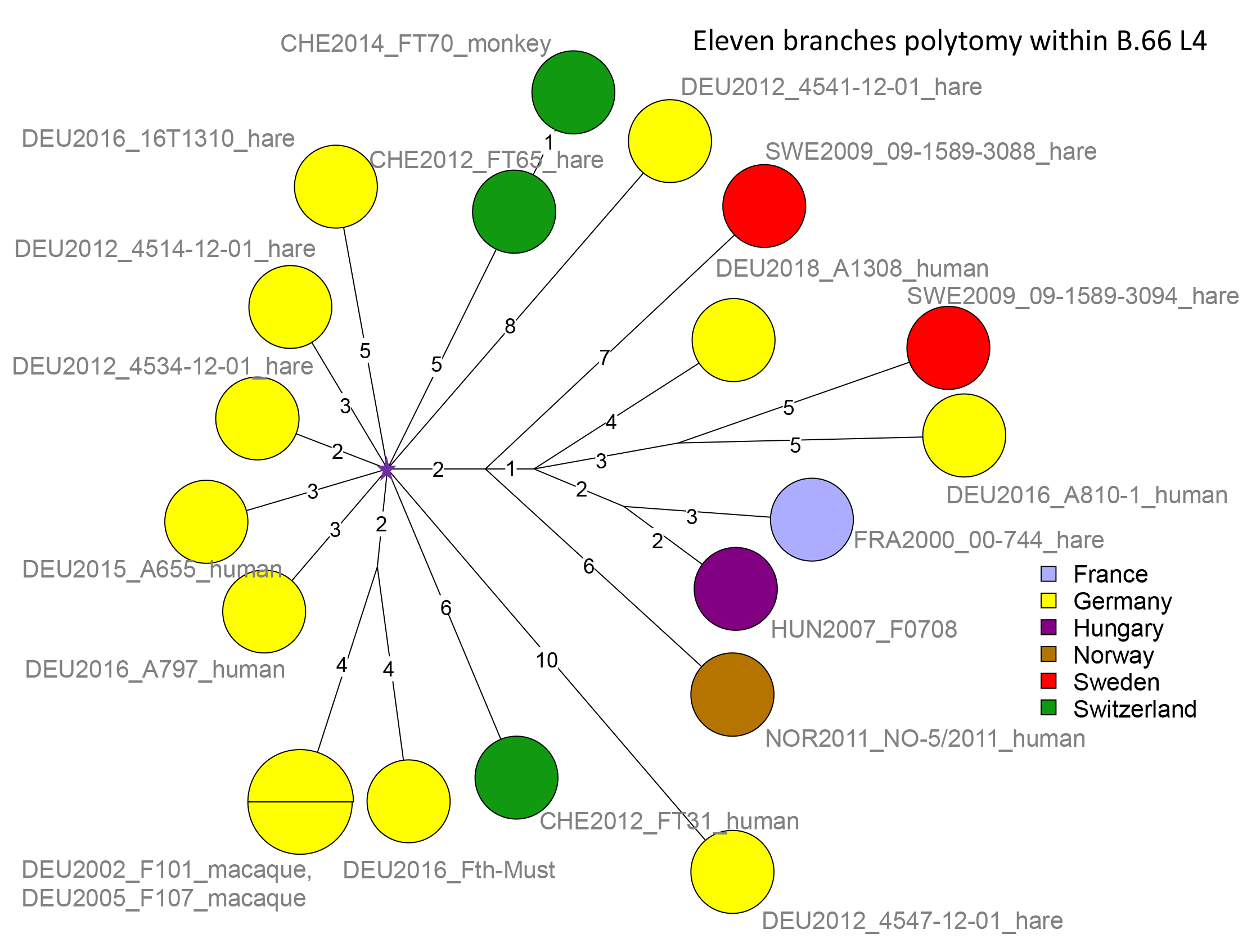

Supplement: S3 Fig — The polytomy comprises 20 strains isolated in Europe, predominantly Germany, in the years 2000-2018. Ninety-six SNPs were identified by mapping on the FSC200 genome sequence (assembly accession GCA_000168775). The Maximum Parsimony tree has a size of 96 (no homoplasy). Branch length from the MRCA indicated by the purple star to the tips varies from two up to eleven SNPs. Circles are labelled with the three-letters country code, year of isolation, strain Id and host. They are colored according to geographic origin. (TIF) [file pntd.0009419.s003.tif]
